# Immune Checkpoint Blockade Therapy for Hepatocellular Carcinoma: Clinical Challenges and Considerations

**DOI:** 10.3389/fonc.2020.590058

**Published:** 2020-10-15

**Authors:** Qi Zhang, Yiwen Chen, Xueli Bai, Tingbo Liang

**Affiliations:** ^1^ Department of Hepatobiliary and Pancreatic Surgery, The First Affiliated Hospital, Zhejiang University School of Medicine, Hangzhou, China; ^2^ Innovation Center for the Study of Pancreatic Diseases of Zhejiang Province, Hangzhou, China; ^3^ Zhejiang Clinical Research Center of Hepatobiliary and Pancreatic Diseases, Hangzhou, China; ^4^ Key Laboratory of Pancreatic Disease of Zhejiang Province, Hangzhou, China

**Keywords:** liver cancer, biomarker, response evaluation, adjuvant therapy, immune checkpoint inhibitor

## Abstract

Although many approaches have been developed for the treatment of hepatocellular carcinoma (HCC) that has both high incidence and high mortality especially in Asian countries, the prognosis of HCC patients is still dismal. Immunotherapy, particularly immune checkpoint inhibitors show encouraging efficacy and have already been widely applied in clinic. However, in contrast to traditional therapies, immunotherapy brings many challenges when using in a real world, including biomarker discovery, response evaluation, adverse event treatment, etc. In this review, we proposed some important and intractable issues in current clinical practice regarding the strategy of immune checkpoint blockade, collected current evidence, and discuss the critical challenges and possible approaches to a bright future.

## Introduction

Although many treatment modalities including hepatic resection, liver transplantation, radiofrequency ablation, transcatheter arterial chemoembolization (TACE), and tyrosine kinase inhibitors have been widely used in clinical practice, the prognosis of hepatocellular carcinoma (HCC) is still dismal. Anti-tumoral immunotherapies especially immune checkpoint inhibitors (ICIs) show encouraging efficacy and shed light on future treatment of HCC. Currently, druggable immune checkpoints include programed cell death protein 1 (PD-1, CD279), programmed death-ligand 1 (PD-L1, CD274), cytotoxic T-lymphocyte-associated protein 4 (CTLA-4, CD152), V-domain Ig suppressor of T cell activation (VISTA), T-cell immunoglobulin and mucin domain-3 (TIM-3), lymphocyte-activation gene 3 (LAG-3), CD40, OX40 (CD134), and 4-1BB (CD137). Since ipilimumab got approved by the US Food and Drug administration (FDA) in 2011, several ICIs have been now used in clinical practice for many solid tumors including HCC. By activating T cells, ICIs ignite natural anti-tumoral potential of these cells and probably lead to more extensive alterations to reverse immunosuppressive tumor microenvironment. With the increasing evidence of clinical application, more mechanisms of ICIs for cancer treatment have been revealed and are far beyond the initial understanding (1).

Early data for the clinical efficacy of ICIs in HCC were mostly from the CheckMate 040 and KEYNOTE-224 trials (both trials testing anti-PD-1 antibodies), which showed an objective response rate of 15–20% as a second-line setting (2, 3). Limited evidence of anti-PD-L1 antibody and anti-CTLA-4 antibody showed an objective response rate (ORR) of 10% and 17.6%, respectively (4, 5). Although ICIs have been frequently used for HCC treatment in the real world, no phase III trial has actually been reported. However, at least 9 phase III trials are currently ongoing, investigating the efficacy of ICIs in various clinical scenarios (**Table 1**). Among all the ICIs, nivolumab from Bristol-Myers Squibb (New York, NY, USA) and camrelizumab from Hengrui (Lianyungang, Jiangsu, China) have been approved as a second-line therapy for HCC patients who had sorafenib refractory or intolerant. However, in real world some late-stage HCC patients received ICIs beyond this indication, and various clinical trials investigating ICIs as a first-line therapy or neoadjuvant therapy are ongoing. Therefore, a considerable number of HCC patients treated by ICIs have been accumulating. Given the mild efficacy of ICIs alone for HCC, combination with other agents such as another ICI, lenvatinib, and apatinib is being explored. ICIs combined with TACE or radiotherapy is also under evaluation. These attempts demonstrate a higher response rate compared to ICI monotherapy.

**Table 1:** Phase III clinical trials of immune checkpoint inhibitors for hepatocellular carcinoma (June 2020). OS, Overall survival; RFS, relapse-free survival; PFS, progression-free survival.

Study name	Treatments	Disease	Line of therapy	Primary outcome	Countries	Study start	Estimated number	Design
CheckMate 459 (NCT02576509)	Nivolumab *vs.* sorafenib	Advanced HCC	First-line	OS	Global	Nov 2015	726	Open label
CheckMate 9DX (NCT03383458)	Nivolumab *vs.* placebo	Postoperative HCC	Adjuvant	RFS	Global	Dec 2017	530	Double blinded
KEYNOTE-240 (NCT02702401)	Pembrolizumab *vs.* placebo	Refractory advanced HCC	Second-line	PFS/OS	Global	May 2016	408	Double blinded
KEYNOTE-394 (NCT03062358)	Pembrolizumab *vs.* placebo	Refractory advanced HCC	Second-line	OS	Asia	Apr 2017	330	Double blinded
KEYNOTE-937 (NCT03867084)	Pembrolizumab *vs.* placebo	Postoperative HCC	Adjuvant	RFS/OS	Global	May 2019	950	Double blinded
NCT03412773	Tislelzumab *vs.* sorafenib	Advanced HCC	First-line	OS	Global	Dec 2017	660	Non-inferiority
HIMALAYA (NCT03298451)	Durvalumab *vs.* durvalumab+tremelimumab *vs.* sorafenib	Advanced HCC	First-line	OS	Global	Oct 2017	1200	Open label
IMbrave150 (NCT03434379)	Atezolizumab+bevacizumab *vs.* sorafenib	Advanced HCC	First-line	OS	Global	Mar 2018	480	Open label
IMbrave050 (NCT04102098)	Atezolizumab+bevacizumab *vs.* active surveillance	Postoperative HCC	Adjuvant	RFS	Global	Dec 2019	662	Open label
NCT03092895	Camrelizumab	Refractory advanced HCC	Second-line	ORR	China	Apr 2017	60	Open label

Undoubtedly, ICIs will play a key role in the treatment modality of HCC. However, as a novel strategy, many critical issues in the clinical scenarios have been emerging and have confused physicians. These issues are also closely related to interpretation of the clinical trials, and thus warranted a deep discussion, which will not only improve the clinical management but also refine the design of future clinical trials.

## Critical Issues in Clinical Practice

### Biomarker Discovery for Prediction of Efficacy of ICIs

Identification of effective biomarkers is critical for the use of ICIs; however, currently there are no ideal ones. For anti-PD-1 and anti-PD-L1 antibodies, the expression of PD-L1 in tumor sections was initially a reasonable biomarker, and the accompanied testing kit of PD-L1 expression was indispensable for approval of an anti-PD-1/PD-L1 antibody by US FDA. Indeed, a higher PD-L1 expression in tumor samples was associated with a higher ORR in the majority of cancers treated by anti-PD-1/PD-L1 antibodies. Whereas, blockade of PD-1/PD-L1 signaling in non-malignant cells was later found also clinically relevant. For instance, PD-1^+^ macrophages within tumors show compromised phagocytosis and impaired tumor immunity (6). In addition, a novel subset of PD-1^high^ regulatory B cell population in HCC was recently uncovered to suppress anti-tumor immunity *via* interleukin (IL)-10 signals (7). Other non-neoplastic cells such as monocytes and mesenchymal stromal cells that express PD-L1 were further proven to inhibit anti-tumor responses and promote cancer progression (8, 9). These results support the rationale PD-1/PD-L1 expression by stromal immune cells as a predictive biomarker for anti-PD-1 or anti-PD-L1 antibodies (10)). Even more, exosomal PD-L1 has been revealed to cause immunosuppression in tumors, and it is believed to be a possible biomarker and therapeutic target for cancer therapy (11). However, more translational studies and clinical trials are warranted for the predictive value of these potential biomarkers in patients treated with ICIs.

It needs great efforts to establish a biomarker in specific scenarios, which include the line of therapy, threshold of protein expression, type of sample (fresh or archival), type of cell staining, kit of companion diagnostic, assay of testing, and particular endpoint for approval (12). For example, PD-L1 protein expression evaluated by immunohistochemistry has been reported unsuitable for prognostic or predictive of benefits from adjuvant chemotherapy in resected non-small cell lung cancer (13). The agreement between immunohistochemistry and other methods such as polymerase chain reaction is not good as shown by the CLOVER comparison study (14). Recently, the posttranslational modification especially the phosphorylation and glycosylation of PD-L1 has been paid much attention and investigators revealed that these modifications significantly affect the detection performance and therapeutic efficacy of PD-L1 antibodies (15, 16). Removal of glycosylation by suitable approaches can boost the positive rate of PD-L1 detection in tumor samples (15). Unfortunately, limited evidence has been accumulated in HCC except that PD-L1 expression by both neoplastic or intratumoral inflammatory cells is related to tumor aggressiveness and suggests clinical benefits when using ICIs targeting PD-1/PD-L1 signaling using a retrospective HCC cohort (17).

Beside PD-L1 expression, other predictive biomarkers for effectiveness of ICIs include immune cell clusters, protein expression, tumor mutational burden (TMB), and gene signatures (18, 19). Our group has divided HCC into three immunophenotypic subtypes (e.g., immunocompetent, immunodeficient, and immunosuppressive) based on their microenvironmental features using CD45 and Foxp3 expression in formalin-fixed paraffin-embedded samples, and proposed different strategies for the use of ICIs in the novel classification system (20). The clinical value of this classification is currently under investigation.

In most cancers including HCC, PD-L1 expression and TMB are independent with each other (21). TMB has been well described as a biomarker of ICIs in a variety of cancers. In HCC, the median TMB is around 4 mutations/Mb, with only approximately 5% of all samples showing a TMB higher than 10 mutations/Mb (22). It has to be noticed that the quantification of TMB is closely related to the methods and kits, which can report distinct values for the same sample. Therefore, comparison of TMB between studies adopting different approaches needs great caution and is usually meaningless. Interestingly, Chinese patients with HCC have a significantly larger part of TMB high compared to Western patients with HCC (9.3% versus 1%) (23). Although higher TMB quantified by whole-genome sequencing, whole-exome sequencing, or the next generation sequencing is positively associated with better survival in patients treated with ICI (24, 25), its clinical value in HCC is still under debate (22, 26). As a minimal invasive and convenient alternative, blood TMB presented good predicted value in some types of cancer (27, 28), but evidence is lacking for HCC. However, it has to be mentioned that although these biomarkers are used with specific cut-off values according to different assays, they are actually continuums as biological characteristics in a population of patients. Thus, the cut-off value may vary based on assays, populations, and types of diseases. Clinical validation is warranted when introducing a biomarker for evaluation of ICI treatment, and clinical interpretation should be made with caution when a biomarker is used with a specific cut-off value in ICI management.

Some potential gene alterations have been revealed to be tightly associated with tumor response to ICIs, and can serve as predictive biomarkers of therapeutic sensitivity to ICIs. Somatic mutations in *RAS* (*KRAS*, *NRAS*, and *HRAS*), *EGFR*, *TP53*, *SMO*, *DDR2*, *FGFR*, *PTCH1*, *MET*, and *PTEN* are frequent and may affect response to ICI treatment [reviewed by Wang et al. (29)]. In addition, germline gene mutations may also predict tumor response to ICIs. For instance, *JAK2* amplification or emergence of *JAK2* at the 9p24.1 site can enhance PD-L1 expression and may result in good response to ICIs (30, 31), while loss-of-function mutations in *JAK1/2* lead to acquired or primary resistance to anti-PD-1 therapy (32). Similarly, genetic alterations down-regulating the interferon signaling such as *IFNGR1*, *IFNGR2*, and *IRF1*, and amplification of genes that inhibit interferon-gamma such as *SOCS1* and *PIAS4* can weaken the efficacy of ICIs (33). These mutations are not rare and should be paid attention in clinical use of ICIs. Thus, the next generation sequencing (NGS)-based gene mutation testing is helpful for a precise choice of ICIs.

Recently, the potential roles of gut microbiota in immunotherapy for tumors have been raised, and gut microbiota may serve as a potential biomarker of ICIs. It is particularly meaningful in HCC due to the natural connection between gut and liver. Previous studies described an intestinal–microbiota–liver axis as the evidence of gut microbiota promoting chronic liver disease progression and hepatocarcinogenesis in patients with advanced liver disease (34, 35). Meanwhile, the crosstalk between microbiota and the immune system at the level of the gut is critical, and there is compelling evidence that the microbiota helps to shape the immune system (36, 37). The impact of the gut microbiota on response to ICIs has been studied since 2005 (38, 39), and increasing studies demonstrated that gut microbiota played in shaping responses to ICIs (40, 41). Routy et al. reported that patients treated with antibiotics for routine indications shortly before, during, or shortly after treatment with anti-PD-1/PD-L1 antibodies had both significantly lower PFS and OS rates compared with patients who had not received antibiotics, suggesting that primary resistance to ICIs may be attributed to abnormal gut microbiome composition (42). Several studies revealed approximately one-third of all patients undergoing anti-CTLA-4 therapy develop intestinal inflammation due to mucosal immune dysregulation (43, 44), suggesting the potential role of gut microbiota in adverse effects of ICIs. Although these investigations were mainly performed on melanoma, a study analyzed fecal samples from HCC patients and found patients responding to immunotherapy showed higher taxa richness and more gene counts than those of non-responders (45). The dynamic variation characteristics of the gut microbiome may provide promising biomarkers and early predictions of the outcomes of ICI treatment in patients with HCC, which may guide disease-monitoring and treatment decision-making. Nevertheless, gut microbiota can be influenced by many environmental, dietary, and lifestyle factors, all of which can potentially affect the immune system and consequentially regulate the response to ICIs (36, 46). Tumor microbiome can lead to an immunosuppressive tumor microenvironment, and its diversity is correlated with overall survival (47, 48). Ablation of bacteria in animal models enhances the efficacy of ICIs by up-regulating PD-1 expression in tumor (47). Unfortunately, it is difficult to perform bacterial ablation in human patients. Therefore, both gut and tumor microbiota as a biomarker of ICI treatment are still far from clinical practice. The role of microbiota, together with other environmental, dietary, and lifestyle factors, in prediction of tumor response to ICIs can be investigated with powerful methods of molecular pathological epidemiology (46, 49).

### Response Evaluation of ICI Treatment

As the use of ICI becomes increasingly available to patients, a major challenge rises, namely, the accurate determination of clinical efficacy. The World Health Organization (WHO) and the Response Evaluation Criteria in Solid Tumors (RECIST) Group have provided standard guidelines to define tumor response to therapy. Whereas these conventional criteria were developed based on data from clinical trials of cytotoxic chemotherapeutic agents for advanced malignancies. These criteria consider therapeutic success as reduction in tumor burden without any new lesions and treatment failure if early tumor growth or appearance of new lesions. Previous studies have confirmed RECIST 1.0 and 1.1 for assessment of therapeutic effectiveness for a wide range of cytotoxic chemotherapeutic agents and these response criteria have been shown to correlate with patient outcomes (50–52).

In the case of HCC, molecular-targeted therapies and therapeutic interventions are main approaches besides surgery. Previous studies have shown a poor correlation between the clinical benefit provided by molecular-targeted therapies such as sorafenib or by locoregional interventional therapies and RECIST assessments, since the antitumor activity in such situations may be presented as tumor necrosis (53, 54). The American Association for the Study of Liver Diseases (AASLD) Practice Guideline on the management of HCC issued in 2005 stated that the evaluation of the treatment response should take into account the induction of intratumoral necrotic areas in estimating the decrease in tumor load (55). The modified RECIST assessment (mRECIST) for HCC was proposed after a series of amendments (56). To be selected as a target lesion using mRECIST, an HCC lesion should meet all the following criteria: 1) The lesion can be classified as a RECIST measurable lesion (i.e., the lesion can be accurately measured in at least one dimension as 1 cm or more). 2) The lesion is suitable for repeat measurement. 3) The lesion shows intratumoral arterial enhancement on contrast-enhanced CT or MRI. In mRECIST, progression disease (PD) is defined as an increase of at least 20% in the sum of the diameters of viable (enhancing) target lesions, taking as reference the smallest sum of the diameters of viable (enhancing) target lesions recorded since treatment started and partial response (PR) is defined as at least a 30% decrease in the sum of diameters of viable (enhancement in the arterial phase) target lesions, taking as reference the baseline sum of the diameters of target lesions. The mRECIST has been validated and widely adopted in subsequent HCC studies.

Nearly all previous and current clinical trials regarding ICI still adopt RECIST 1.1 for standard of response evaluation. However, clinical practice found that in contrast to chemotherapy and targeted therapy, ICI treatment has a considerate rate of hyperprogression and pseudoprogression, leading to dramatically different decision-making based on tumor size. In a cohort of East Asian patients with HCC, up to 23% patients who received PD-1 blockade were reported to suffer from hyperprogressive disease (HPD) (57). In this real-world study, neutrophil-to-lymphocyte ratio was the only identified biomarker to predict HPD, suggesting to avoid using ICIs in such patients.

Clinical observation revealed that some patients responded to ICIs with tumor shrinkage or stable disease that was consistent with RECIST criteria; however, distinct immune-related patterns of response have been noted, including development of new lesions associated with edema and infiltration of immune cells and transient increase in the size of primary lesions (58). Delayed clinical responses such as an increase in total tumor burden were followed by significant tumor regression. Experience from patients with melanoma treated with ipilimumab indicated that the initially enlarged lesions could be infiltrated by massive inflammatory cells and necrotic tumor cells, in patients with subsequent decreased tumor burden (59, 60), which induced the definition of pseudoprogression. Pseudoprogression is defined as more than 25% increase in tumor burden at week 12 (early) or any assessment after week 12 (delayed) that was not confirmed as progressive disease at next assessment. These findings of pseudoprogression would have been classified prematurely as progressive disease by WHO or RECIST criteria and have prompted the development of the immune-related response criteria (irRC). Actually, several clinical trials reported that a few patients showed distinct immune-related patterns of treatment response that did not meet RECIST criteria (61), including the clinical trials in our center. Based on survival analysis, conventional RECIST might underestimate the benefit of pembrolizumab in approximately 15% of patients with advanced melanoma in the phase Ib KEYNOTE-001 study (61). The irRC was first proposed based on data from a phase 2 clinical trial of ICIs in patients with melanoma (58). The irRC resembles the conventional criteria for determination of overall tumor burden at baseline, which includes selection of both measurable (target/index) and non-measurable (non-target/non-index) lesions with similar standards. While in irRC, PD is defined as at least 25% increase in tumor burden compared with nadir (at any single time point) in two consecutive observations at least 4 weeks apart. The irRC has been externally validated in melanoma and non-small lung cancer from the perspective of their association with outcomes.

Since robust responses have been recorded with ICI in HCC, various clinical trials have been carried out to explore the indications and combinations of ICIs in HCC. In KEYNOTE-224, investigators compared the response evaluation results according to RECIST 1.1, mRECIST, and irRC, and found similar results among the three criteria (3). While in this study, patients only received pembrolizumab. Whether the three criteria have a similar evaluation performance in other ICIs or in combination modality regarding ICIs especially when locoregional interventional therapies were combined is unclear. Future studies are urgently needed to validate since the evaluation criteria may deeply influence the clinical practice and judgment of results from clinical trials.

### On-Target, Off-Tumor Effects for ICI Therapy

Unintended auto-immune complications can occur when the immune system is enhanced to fight cancer. Adverse events of ICI include colitis, endocrinopathies such as hypophysitis and thyroid disorders, or type 1 diabetes mellitus, hepatitis presented as increased aspartate aminotransferase concentration, alanine aminotransferase concentration, elevated bilirubin concentration, or cholestatic jaundice, pancreatitis, pneumonitis, dermatitis, and/or sarcoid-like reaction. These immune-related adverse events can occur at any stage of ICI therapy. In particular, whether previous hepatic diseases and hepatitis viruses infection increase ICI-associated hepatitis needs further study. The median onset of events typically ensues during the following time periods: varies by organ system affected with-skin-related events at 3 weeks, hepatitis at 3 to 9 weeks, gastrointestinal manifestations at 8 weeks, and endocrinopathies at 7 to 20 weeks. Immune-related adverse events can be managed according to NCCN guidelines (Version 2.2019). Almost all of these immune-related adverse events can be treated by stopping the immune-therapy and administering steroids.

Hyperprogression of HCC can be recognized as a special adverse event, and is characterized by rapid increase in tumor burden in patients treated with immune-therapy. Champiat et al. noted hyperprogression in 9% of patients treated with ICIs (62). In cases of hyperprogression, characteristics of progression include time to treatment failure less than 2 months, more than 50% increase in tumor burden compared to pre-baseline levels and more than two-fold increase in pace of tumor growth (63). The patients with HPD usually have a deteriorating clinical condition and lead to treatment failure. Sonja Kleffel et al. reported that PD-1/PD-L1 signaling has cell-intrinsic functions in tumor cells (64). It is possible that PD-1/PD-L1 blockade might affect alternative signaling pathways and accelerate tumor growth and tumorigenesis. Several genetic alterations (e.g., KRAS and STK11 mutations, MDM2, MDM4 and EGFR amplifications) have been reported to be associated with ICI-related HPD (65, 66). The rapid proliferation of PD-1^+^ effector regulatory T cells after ICI treatment was found to promote HPD in patients with gastric cancer (67). Older age, higher metastatic burden, and previous radiation are found associated with HPD (68). Many studies have explored potential biomarkers from clinical, laboratory, and imaging angles (69). Unfortunately, there are no reliable ways to select patients with HCC who are risky for HPD before the treatment of ICIs.

### Choice of Immunotherapy for Patients With HCC

Although there are many ICIs such as anti-LAG3 and anti-PD-L2 under development nowadays, only anti-PD-1, anti-PD-L1, and anti-CTLA4 antibodies have been approved for clinical use. These include three anti-PD-1 antibodies (nivolumab, pembrolizumab, cemiplimab), three anti-PD-L1 antibodies (atezolizumab, avelumab, durvalumab), and one anti-CTLA4 antibody (ipilimumab). In China, another four PD-1 antibodies (sintilimab, camrelizumab, tislelizumab, and toripalimab) were approved with specific indications. Merely nivolumab, pembrolizumab, atezolimumab, and camrelizumab have approved indications for HCC in different regions of the world. However, off-label use of these ICIs is quite common in the real world. The overall estimation of off-label use of ICIs in all cancers is between 18% and 30% (70), and HCC was once the commonest disease (more than a half) that was treated by ICIs as the off-label approach (71).

The CTLA4 and PD-1 pathways are different in human immunity, with CTLA4 regulating T cell proliferation in the early stage of immune response while PD-1 suppressing T cells in the late stage of immune response (72). CTLA4 is restricted to antigen-presenting cells and PD-1 is related to not only immune cells but also tumor cells (1). Given the differences of the two signals, combination of anti-CTLA4 and anti-PD-1/PD-L1 antibodies is reasonable and approved by US FDA in melanoma, but evidence is lacking for the combinatory use of ICIs in HCC. Since HCC cells express extensive PD-L1, strategies to block PD-1/PD-L1 signal are more acceptable than anti-CTLA4 therapy. Although *in vitro* functional assays demonstrated that currently available therapeutic PD-L1 antibodies are more superior to PD-1 antibodies in blocking PD-1/PD-L1 signaling (73), a systematic review and meta-analysis showed that anti-PD-1 antibodies were generally better than anti-PD-L1 antibodies in terms of both overall survival (HR 0.75) and progression-free survival (HR 0.73) for solid tumors (74). This may be because anti-PD-L1 antibody does not block PD-L2 induced PD-1 signal in T cells, and PD-L2 is also overexpressed and performs as a prognostic factor for HCC (75). Although some antibodies have special designs to minimize side effects and optimize efficacy, most physicians from their limited clinical observations believe that the efficacy and adverse events are similar among them since there are no head-to-head trials comparing the efficacy among these ICIs. Therefore, no evidence is available to recommend a certain ICI in HCC management.

### Immunotherapy as a (Neo)Adjuvant Therapy for Resectable HCC

Rapid recurrence of HCC after curative resection or ablation is an unmet medical need. Compared to late recurrence that is sometimes believed to be independent carcinogenesis especially for patients with hepatitis B virus infection or liver cirrhosis, patients at risk for early recurrence based upon tumor characteristics may be ideal to receive immediate adjuvant immunotherapy to eliminate or control residual, perhaps radiologically occult, tumor cells. Up to date, many strategies has been explored to try to prevent or delay postoperative recurrence using TACE, sorafenib, and Huaier granule (76–78). In contrast to the weak effectiveness of TACE and sorafenib, which target tumor-associated microvessels and/or tumor cells themselves, ICIs may be more reasonable to be applied to reduce recurrence rate after surgery or ablation because tumor cells are removed but tumor-associated antigens are exposed. Recently, the use of ICIs for adjuvant therapy has been discussed. Although there is no solid evidence to support using ICI in such clinical scenario, a randomized phase III trial (IMbrave050, NCT04102098) testing atezolizumab plus bevacizumab in patient with HCC at high risk of recurrence after curative treatment was launched. Another phase III trial (KEYNOTE-937, NCT03867084) comparing pembrolizumab and placebo as adjuvant therapy in patients with HCC and complete radiological response after surgical resection or local ablation is also recruiting participants. A clinical trial with similar design for HCC patients at high risk of recurrence is ongoing for nivolumab (CheckMate 9DX, NCT03383458). In our center, a similar clinical trial as adjuvant therapy is ongoing for toripalimab and donafenib (CISLD-8, NCT04418401). There is no standard duration of adjuvant therapy for HCC, and in existing clinical trials mentioned above, the adjuvant therapy lasts for 6 to 12 months.

Another approach to decrease the recurrence rate of HCC after curative surgery is neoadjuvant therapy. Neoadjuvant approach with immune-based therapies may prove to be successful because tumor antigens are more available before eradication of the tumor by surgery. Neoadjuvant application of pembrolizumab was safe and efficacious in patients with NSCLC (79). So far, there is no approved indications of neoadjuvant treatment of ICI. For HCC, neoadjuvant immunotherapy using ICI has just been initiated. In 2020 ASCO, a randomized phase II pilot trial evaluating nivolumab alone or nivolumab plus ipilimumab in patients with resectable HCC reached its primary endpoint of safety (NCT03222076). In this study, researchers reported a pathologic response rate of 40% (24% pCR and 16% major necrosis) for resectable HCC after preoperative immunotherapy (80). There are also several clinical trials for advanced HCC as neoadjuvant therapy that aim for down staging to reach the criteria for curative surgery. The combination of transarterial radioembolization (TARE) with nivolumab is being studied in the neoadjuvant setting (NCT03812562). Another clinical trial as neoadjuvant therapy is the combination of drug-eluting TACE and sintilimab (CISLD-5, NCT04174781). For HCC patients waiting for liver transplantation, there are no available data but there is an ongoing clinical trial testing the combination of camrelizumab and apatinib for downstaging or bridging before liver transplantation (NCT04035876). These clinical trials are highly anticipated and highlight the potentially important role of immune checkpoint blockade therapy in preoperative treatment in HCC.

### Timing of Introducing Immunotherapy for Patients With Unresectable HCC

Currently only three RCTs (CheckMate-459, KEYNOTE-240, and IMbrave 150) with available results assess the clinical efficacy of ICIs compared with the standard of care in unresectable HCC patients. In first-line setting, nivolumab and atezolizumab beat sorafenib in terms of OS and ORR (81). Several trials with monotherapy of ICI or combination therapy of ICI and other treatment are ongoing (**Table 1**). Taking the advantage of similar genomic characteristics of tumor nodules in multifocal HCC patients, Huang et al. revealed that small tumors had higher immune cell infiltration and better sensitivity to anti-PD-1 therapy compared with large tumors (82). These results support early use of ICI in HCC patients without opportunity for radical resection. Intriguingly, PD-L1 expression in infiltrating macrophages rather than tumor cells was found up-regulated in patients with HCC and resistant to sorafenib treatment; additionally, circulating soluble PD-L1 was also increased (83, 84). These evidences may provide rationale for the use of PD-1/PD-L1 blockade as a second-line therapy.

### Approaches to Enhance the Efficacy of ICI in Patients With HCC

The objective response rate (ORR) of ICI alone is not clinically satisfactory; thus, physicians have been investigating combination strategies to enhance the efficacy of ICIs. The US FDA has approved the combination regimen of atezolizumab and bevacizumab for advanced HCC according to a phase 1b trial, which demonstrated an ORR of 34% (RECIST1.1) and 25% of patients suffered from adverse events with grade 3 or higher (85). Another phase 1b trial testing the combinatory use of pembrolizumab and lenvatinib showed an ORR of 46% and a DCR of 92% (mRECIST) (86). Retrospective evaluation of CheckMate-040 showed that nivolumab with local-regional treatment group had an ORR of 50%, a CR of 11%, and median OS of 13.6 months, which were far better than the general results reported by the original study (2, 87). Two ICIs targeting PD-L1 and CTLA-4, respectively, are also used together in studies of advanced HCC. Durvalumab and tremelimumab combination therapy led to an ORR of 18% and a DCR of 57.5% with 20% of patients had grade 3/4 treatment-related adverse events, showing minimal enhancement of efficacy and increased risk of severe side effect compared to one ICI alone (88). Similarly, tremelimumab plus durvalumab showed an ORR of 20% and a DCR of 60% in advanced HCC with median PFS of 7.8 months (89).

Hundreds of clinical trials have been developed to test different agents including current available drugs and novel chemicals. Among these agents, anti-angiogenic molecules are currently promising and have been proved by several key trials. For instance, based on the optimal results of IMbrave 150 study, which showed reduced risk of death by 42% in the study group when compared with sorafenib, combination of atezolizumab and bevacizumab has been approved by US FDA to treat patients with unresectable or metastatic HCC (90). Other anti-angiogenic agents and multi-kinase inhibitors such as lenvatinib, ramucirumab, nintedanib, sorafenib, axitinib, and capmatinib have been under investigation in patients with HCC (91). Using mouse models, people revealed that such combination can induce high endothelial venules that promote cytotoxic T cell infiltration, activity, and consequent tumor cell destruction (92). In addition, agents targeting c-Met and TGF-β receptor I are also being tested for their ability to enhance ICI treatment in patients with HCC (NCT02423343 and NCT02795429).

There are some other promising combination strategies that are currently explored in other solid tumors in clinical trials or preclinical studies. A DDR inhibitor AZD6738 and radiotherapy combined with anti-PD-L1 antibodies could perform better tumor growth inhibition and recurrence prevention by boosting CD8^+^ T cell infiltration and activation in tumor microenvironment in a mouse model (93). However, these strategies are still in preclinical stage or under *in vivo* testing, and need time and luck to be translated into clinic.

## Perspectives

With the increasing cases of HCC receiving ICI treatment, physicians are gaining more and more experience, while more problems are arising. Some of them are pan-cancer associated, and some of them are HCC specific. ICI is becoming a mainstay of the comprehensive management of HCC, and these clinical challenges need well-designed clinical studies to conquer. Before we get the answers, careful use of ICIs within indications or as an off-label way should balance its benefits and risks.

## Author Contributions

QZ and YC contributed equally to this work. QZ and TL conceived the idea. QZ and YC wrote the draft. TL and XB interpreted the clinical significance. All authors contributed to the article and approved the submitted version.

## Funding

This work was financially supported by the National Natural Science Foundation of China (81871320 to QZ and 81830089 to TL), Zhejiang Provincial Natural Science Foundation of China (LR20H160002 to QZ), and Zhejiang Provincial Program for the Cultivation of High-level Innovative Health Talents (to XB).

## Conflict of Interest

The authors declare that the research was conducted in the absence of any commercial or financial relationships that could be construed as a potential conflict of interest.
